# Real‐world clinical experience with serum MOG and AQP4 antibody testing by live versus fixed cell‐based assay

**DOI:** 10.1002/acn3.52310

**Published:** 2025-02-03

**Authors:** Yana Said, Angeliki Filippatou, Conlan Tran, LuAnn Rezavi, Kai Guo, Matthew D. Smith, Yasmin Resto, John J. Chen, Peter A. Calabresi, Patrizio Caturegli, Sean J. Pittock, Eoin P. Flanagan, Elias S. Sotirchos

**Affiliations:** ^1^ Department of Neurology Johns Hopkins University School of Medicine Baltimore Maryland USA; ^2^ Department of Pathology Johns Hopkins University School of Medicine Baltimore Maryland USA; ^3^ Departments of Neurology, Laboratory Medicine and Pathology, and Center for MS and Autoimmune Neurology Mayo Clinic College of Medicine Rochester Minnesota USA; ^4^ Departments of Ophthalmology and Neurology, Center for MS and Autoimmune Neurology Mayo Clinic Rochester Minnesota USA

## Abstract

**Objective:**

To assess the real‐world performance of a live (LCBA) versus a fixed (FCBA) cell‐based assay for the detection of serum antibodies directed against myelin oligodendrocyte glycoprotein (MOG‐IgG) and aquaporin‐4 (AQP4‐IgG).

**Methods:**

This was a retrospective study of patients evaluated at a single tertiary academic referral center, with serum testing performed clinically for AQP4‐IgG and/or MOG‐IgG by FCBA and LCBA on the same day. Additionally, frozen banked sera from the same day for patients tested only by one assay were retrieved and tested by the other assay. FCBA was performed by the Johns Hopkins Immunology Laboratory using Euroimmun kits with detection by indirect immunofluorescence (FCBA‐IF), whereas LCBA was performed by the Mayo Clinic Neuroimmunology Laboratory with detection by flow cytometry (LCBA‐FACS).

**Results:**

Of 594 specimens with paired MOG‐IgG testing, 500 were negative by both assays, 33 were positive by both assays, 56 were positive exclusively by LCBA‐FACS, and 5 were only positive by FCBA‐IF. Overall, MOG‐IgG LCBA‐FACS exhibited 95.1% sensitivity and 97.7% specificity, whereas MOG‐IgG FCBA‐IF had 45.7% sensitivity and 99.8% specificity. Of 577 specimens with paired AQP4‐IgG testing, 503 were negative by both assays, 51 were positive by both assays, 21 were positive exclusively by LCBA‐FACS, and 2 were only positive by FCBA‐IF. Overall, AQP4‐IgG LCBA‐FACS exhibited 97.3% sensitivity and 100% specificity, whereas AQP4‐IgG FCBA‐IF had 71.6% sensitivity and 100% specificity.

**Interpretation:**

LCBA‐FACS for both MOG‐IgG and AQP4‐IgG had markedly better sensitivity than FCBA‐IF, with similar specificity. The use of FCBA‐IF may result in underrecognition of both MOG antibody‐associated disease (MOGAD) and AQP4‐IgG seropositive neuromyelitis optica spectrum disorder (NMOSD).

## Introduction

Myelin oligodendrocyte antibody‐associated disease (MOGAD) and aquaporin‐4 antibody (AQP4‐IgG) seropositive neuromyelitis optica spectrum disorder (AQP4+ NMOSD) are auto‐antibody‐associated CNS inflammatory diseases.^1^, ^2^ Their diagnosis relies on the detection of serum MOG‐IgG and AQP4‐IgG, respectively, in patients with compatible clinical phenotypes.

While live cell‐based assays (LCBA) are the gold standard for the detection of MOG‐IgG and AQP4‐IgG, fixed cell‐based assays (FCBA) are more widely accessible due to technical issues and cost.^3^ However, discrepancies in performance between LCBA and FCBA have been found in some comparative studies, especially for MOG‐IgG testing, while other studies have reported good agreement of FCBA and LCBA.^3^, ^4^, ^5^, ^6^, ^7^, ^8^, ^9^, ^10^, ^11^, ^12^ Comparative studies have varied in many aspects, including the specific assays utilized, the setting (research vs. routine clinical testing), the inclusion of a single or multiple centers, the underlying study population characteristics, and the availability of clinical data.

In this study, we sought to assess the real‐world performance of LCBA versus FCBA testing for serum MOG‐IgG and AQP4‐IgG in patients evaluated at a single tertiary academic referral center, utilizing the two most widely utilized and available commercial CBAs in the United States.

## Methods

### Study population and laboratory assays

The study protocol was approved by the Johns Hopkins University Institutional Review Board (IRB00416716). We retrospectively identified all patients from Johns Hopkins with clinical MOG‐IgG or AQP4‐IgG testing between July 2019 and January 2024 performed by the Johns Hopkins Immunology Laboratory (FCBA) and the Mayo Clinic Neuroimmunology Laboratory (LCBA) on serum specimens collected on the same day. FCBAs were performed utilizing Euroimmun kits (Lübeck, Germany) according to the manufacturer's instructions, with detection by indirect immunofluorescence (IF).^13^, ^14^ LCBAs were performed with detection by flow cytometry (FACS), as previously described.^3^, ^15^ These assays use secondary antibodies directed against human IgG (detecting all IgG subclasses), except for the LCBA‐FACS assay for MOG, which uses an IgG‐1 specific secondary antibody.

The typical clinical scenarios for testing of specimens by both assays were as follows: (1) the ordering physician would order FCBAs and LCBAs simultaneously in patients with high suspicion for MOGAD or NMOSD; (2) residual serum stored at 4°C from MOG‐IgG and/or AQP4‐IgG testing at Johns Hopkins could be requested by the ordering physician within 28 days of collection to be sent to the Mayo Clinic for testing (e.g., to confirm a positive result or in cases with high clinical suspicion but negative testing locally).

Furthermore, we identified visits at which testing was performed clinically by only one assay for either MOG‐IgG or AQP4‐IgG, with banked frozen serum specimens (−80°C) collected on the same day for long‐term storage in a research bio‐repository. Samples were retrieved and sent for testing by the other assay, with laboratory staff at both locations masked to patient information.

Diagnoses of AQP4+ NMOSD and MOGAD were assessed according to the 2015 International Panel for NMO Diagnosis (IPND) criteria and the 2023 MOGAD diagnostic criteria, respectively.^1^, ^2^ MOG‐IgG titers of ≥1:100 were considered clear‐positive for both LCBA‐FACS and FCBA‐IF, in accordance with published proposed cut‐offs.^1^ Patients with low MOG‐IgG titers were required to fulfill supportive clinical or MRI criteria, in accordance with the MOGAD diagnostic criteria.^2^

### Data analysis

Statistical analysis was performed using R version 4.3.2. Kendall's tau rank correlation was used to assess correlation of positive titers between assays. For the primary analysis, false‐negative results were defined for each assay as negative results that were positive by the other assay in patients fulfilling diagnostic criteria for MOGAD or AQP4+ NMOSD. Notably, this approach is distinct from defining sensitivity using clinical NMOSD diagnosis (regardless of AQP4‐IgG serostatus) or diagnosis based on historical MOG/AQP4‐IgG serostatus, as the goal was to compare the two assays' performance and as seroreversion may occur in these conditions spontaneously (especially in MOGAD) or in the setting of immunosuppressive therapy.^15^, ^16^, ^17^ We also performed secondary analyses considering patients fulfilling AQP4+ NMOSD or MOGAD diagnostic criteria based on historical AQP4‐IgG or MOG‐IgG seropositivity who were now negative by both assays (seroreversion) as being “false‐negatives.” Sensitivity was calculated as true‐positives/(true‐positives + false‐negatives), whereas specificity was calculated as true‐negatives/(true‐negatives + false‐positives), with their respective confidence intervals calculated using the Clopper–Pearson method.

## Results

**Figure 1:**
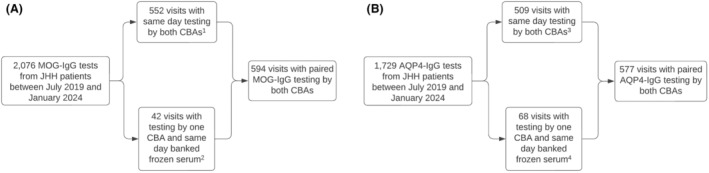
Flowchart of sample selection process for inclusion in the study for MOG‐IgG (A) and AQP4‐IgG (B). *(1) 489 negative samples by both CBAs, 17 positive samples by both CBAs, 45 positive samples by LCBA‐FACS only, and 1 positive sample by FCBA only. (2) 11 negative samples by both CBAs, 16 positive samples by both CBAs, 11 positive samples by LCBA only, and 4 positive samples by FCBA only. (3) 487 negative samples by both CBAs, 9 positive samples by both CBAs, 12 positive samples by LCBA only, and 1 positive sample by FCBA only. (4) 16 negative samples by both CBAs, 42 positive samples by both CBAs, 9 positive samples by LCBA only, and 1 positive sample by FCBA only.*

Figure 1 outlines the process for participant/specimen selection for inclusion in the study.

### MOG‐IgG assay performance

**Figure 2:**
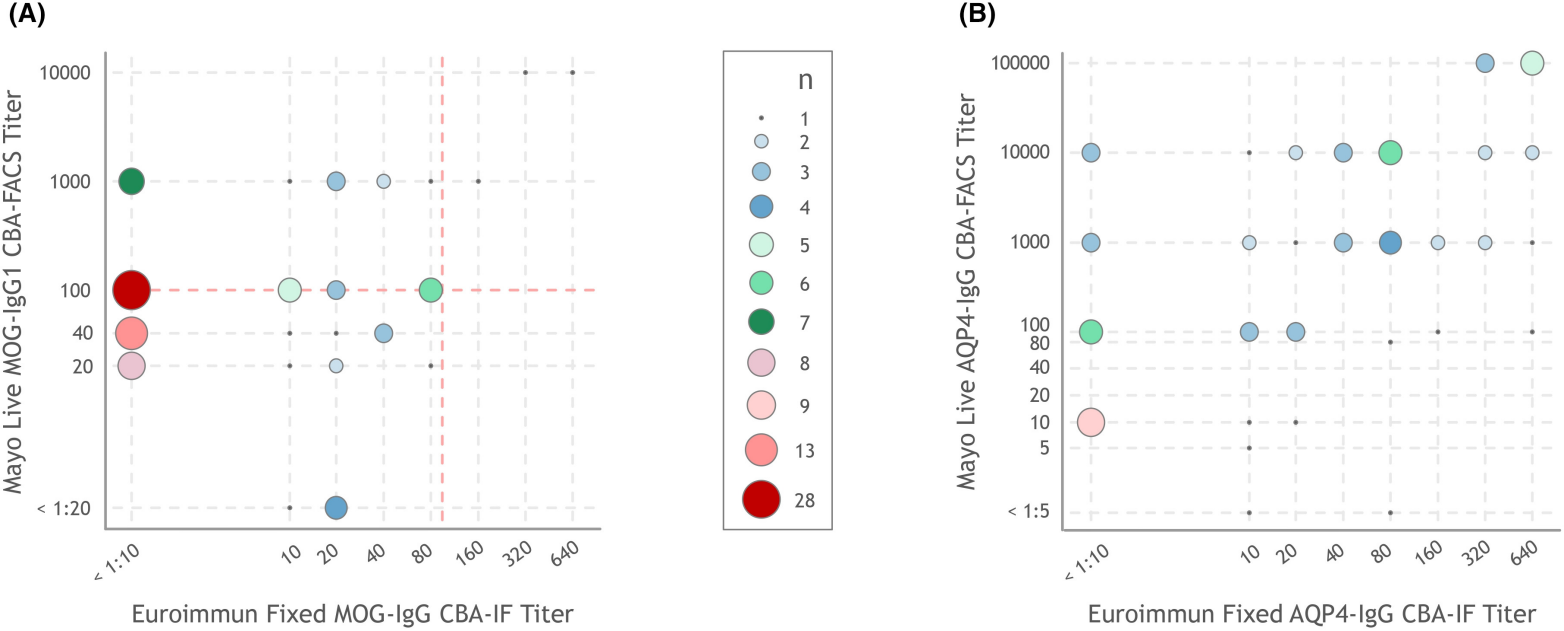
Comparison of (A) MOG‐IgG and (B) AQP4‐IgG titers by FCBA‐IF versus LCBA‐FACS among samples testing positive by at least one CBA. FCBA‐IF, fixed cell‐based assay using immunofluorescence; IgG, immunoglobulin; LCBA‐FACS, live cell‐based assay using flow cytometry. The red dashed line in the MOG‐IgG titer (A) represents the cut‐offs for clear‐positive versus low‐positive titers (1:100 dilution for both assays).

Of 594 specimens with paired MOG‐IgG testing (563 unique patients), 500 were negative by both CBAs, 33 were positive by both, 56 were positive exclusively by LCBA‐FACS, and 5 were only positive by FCBA‐IF (Fig. 2A, Table 1, Fig. S1, Table S1). Overall, MOG‐IgG LCBA‐FACS exhibited 95.1% (95% CI: 88%–99%) sensitivity and 97.7% (95% CI: 96%–99%) specificity, whereas MOG‐IgG FCBA‐IF had 45.7% (95% CI: 45%–57%) sensitivity and 99.8% (95% CI: 99%–100%) specificity (Tables 2 and 3). Of the 33 specimens (27 unique patients) that tested positive by both assays, 24 (73%) were clear‐positive by LCBA‐FACS, whereas only 3 (9%) were clear‐positive by FCBA‐IF. However, all patients positive by both assays met diagnostic criteria for MOGAD when utilizing either the LCBA‐FACS or FCBA‐IF titers (with supporting clinical features for those with low‐positive titers). Notably, 16 of the 33 samples were collected <90 days after the onset of an attack, with 15 of 16 (94%) having clear‐positive titers by LCBA‐FACS and only 3 of 16 (19%) by FCBA‐IF. Correlation of positive MOG‐IgG titers between the two assays was not statistically significant (*τ* = 0.24; *P* = 0.1).

**Table 1:** Comparison of MOG‐IgG results between FCBA‐IF and LCBA‐FACS.

	MOG‐IgG LCBA‐FACS			
	Result	Positive	Negative	Total
MOG‐IgG FCBA‐IF	Positive	33	5	38
	Negative	56	500	556
	Total	89	505	594

FCBA‐IF, fixed cell‐based assay using immunofluorescence; IgG, immunoglobulin; LCBA‐FACS, live cell‐based assay using flow cytometry.

**Table 2:** Contingency table of MOG‐IgG LCBA‐FACS and MOGAD diagnosis.

	MOGAD diagnosis	No MOGAD diagnosis or seroreversion by both assays	Total	MOGAD diagnosis	No MOGAD diagnosis	Total
MOG‐IgG LCBA‐FACS positive	77	12	89	77	12	89
MOG‐IgG LCBA‐FACS negative	4	501	505	22	483	505
Total	81	513	594	99	495	594

FCBA‐IF, fixed cell‐based assay using immunofluorescence; IgG, immunoglobulin; LCBA‐FACS, live cell‐based assay using flow cytometry.

**Table 3:** Contingency table of MOG‐IgG FCBA‐IF and MOGAD diagnosis.

	MOGAD diagnosis	No MOGAD diagnosis or seroreversion by both assays	Total	MOGAD diagnosis	No MOGAD diagnosis	Total
MOG‐IgG FCBA‐IF positive	37	1	38	37	1	38
MOG‐IgG FCBA‐IF negative	44	512	556	62	494	556
Total	81	513	594	99	495	594

FCBA‐IF, fixed cell‐based assay using immunofluorescence; IgG, immunoglobulin; LCBA‐FACS, live cell‐based assay using flow cytometry.

The 56 specimens exclusively MOG‐IgG seropositive by LCBA‐FACS (35 clear‐positive titer; 63%) corresponded to 48 unique patients, of whom 36 (75%) met diagnostic criteria for MOGAD. Clear‐positive titers were present in 31 of 44 (70%) specimens from patients with a diagnosis of MOGAD. Notably, 6 MOGAD patients had paired testing by the two assays on ≥2 occasions, with repeatedly negative testing by FCBA‐IF and consistently positive testing by LCBA‐FACS. Among samples collected <90 days from an attack from MOGAD patients (15 total samples), 11 of 15 (73%) had clear‐positive titers. Among the 12 patients (12 specimens) exclusively positive by LCBA‐FACS who did not meet the criteria for MOGAD, 4 (33%) had clear‐positive titers (1:100 for all). Alternative clinical diagnoses in this group (Table S3) included multiple sclerosis (*n* = 4), clinically isolated syndrome (*n* = 1), radiologically isolated syndrome (*n* = 2), migraines (*n* = 1), biopsy‐confirmed giant cell arteritis (*n* = 1), meningoencephalitis due to lymphocytic choriomeningitis virus (*n* = 1), nutritional optic neuropathy (*n* = 1), and epidural lipomatosis (*n* = 1).

The five specimens solely positive by fixed CBA had low end‐titers (≤1:20) and corresponded to five unique patients, of whom four fulfilled diagnostic criteria for MOGAD and 1 was diagnosed with functional vision loss (Table S3). Of these four MOGAD patients, two had historically tested positive by LCBA‐FACS (both with titers of 1:40), while the remaining two had never previously been tested by LCBA‐FACS. Furthermore, at the time of blood collection, all four MOGAD patients were at >6 months since their last attack and were on chronic immunosuppressive maintenance treatment (one mycophenolate mofetil, one methotrexate, one rituximab, and one oral prednisone).

Overall, the total number of samples drawn from MOGAD patients within 90 days of an attack was 31 (15 exclusively positive by LCBA‐FACS and 16 positive by both assays). Thus, in the acute setting, MOG‐IgG LCBA‐FACS had a sensitivity of 100% (95% CI: 89%–100%) (31/31), whereas MOG‐IgG FCBA‐IF had 51.6% (95% CI: 33%–70%) (16/31) sensitivity. A secondary analysis was performed considering patients fulfilling MOGAD diagnostic criteria based on historical MOG‐IgG seropositivity who were now negative by both assays (seroreversion) as being “false‐negatives.” Using this method, MOG‐IgG LCBA‐FACS exhibited 77.8% (95% CI: 68%–86%) sensitivity and 97.6% (95% CI: 96%–99%) specificity, whereas MOG‐IgG FCBA‐IF had 37.4% (95% CI: 28%–48%) sensitivity and 99.8% (95% CI: 99%–100%) specificity (Tables 2 and 3).

No differences in clinical characteristics (attack phenotype, relapsing vs. monophasic course) or demographic characteristics (age and sex) were observed between patients testing positive by both MOG‐IgG FCBA‐IF and LCBA‐FACS and patients testing positive by only LCBA‐FACS.

### AQP4‐IgG assay comparison

Of 577 specimens with paired AQP4‐IgG testing (545 unique patients), 503 were negative by both CBAs, 51 were positive by both, 21 were positive exclusively by LCBA‐FACS, and 2 were only positive by FCBA‐IF (Fig. 2B, Table 4, Table S2). All patients who tested positive for AQP4‐IgG fulfilled the 2015 IPND diagnostic criteria for AQP4+ NMOSD. Overall, AQP4‐IgG LCBA‐FACS exhibited 97.3% (95% CI: 91%–100%) sensitivity and 100% (95% CI: 99%–100%) specificity, whereas AQP4‐IgG FCBA‐IF had 71.6% (95% CI: 60%–81%) sensitivity and 100% (95% CI: 99%–100%) specificity (Tables 5 and 6). The correlation of positive AQP4‐IgG titers between the two assays was moderate (*τ* = 0.46; *P* < 0.001).

**Table 4:** Comparison of AQP4‐IgG results between FCBA‐IF and LCBA‐FACS.

	AQP4‐IgG LCBA‐FACS			
	Result	Positive	Negative	Total
AQP4‐IgG FCBA‐IF	Positive	51	2	53
	Negative	21	503	524
	Total	72	505	577

FCBA‐IF, fixed cell‐based assay using immunofluorescence; IgG, immunoglobulin; LCBA‐FACS, live cell‐based assay using flow cytometry.

**Table 5:** Contingency table of AQP4‐IgG LCBA‐FACS and NMOSD diagnosis.

	NMOSD diagnosis	No NMOSD diagnosis or seroversion by both assays	Total	NMOSD diagnosis	No NMOSD diagnosis	Total
AQP4‐IgG LCBA‐FACS positive	72	0	72	72	0	72
AQP4‐IgG LCBA‐FACS negative	2	503	505	33	472	505
Total	74	503	577	105	472	577

FCBA‐IF, fixed cell‐based assay using immunofluorescence; IgG, immunoglobulin; LCBA‐FACS, live cell‐based assay using flow cytometry.

**Table 6:** Contingency table of AQP4‐IgG FCBA‐IF and NMOSD diagnosis.

	NMOSD diagnosis	No NMOSD diagnosis or seroversion by both assays	Total	NMOSD diagnosis	No NMOSD diagnosis	Total
AQP4‐IgG FCBA‐IF positive	53	0	53	53	0	53
AQP4‐IgG FCBA‐IF negative	21	503	524	52	472	524
Total	74	503	577	105	472	577

FCBA‐IF, fixed cell‐based assay using immunofluorescence; IgG, immunoglobulin; LCBA‐FACS, live cell‐based assay using flow cytometry.

Of the 21 samples (20 unique patients) exclusively AQP4‐IgG seropositive by LCBA‐FACS, 4 were drawn within 90 days of an acute attack. The two samples exclusively AQP4‐IgG seropositive by FCBA‐IF were drawn from two unique patients during periods of remission, of whom one was untreated and one was under treatment with mycophenolate mofetil and had historically tested positive for AQP4‐IgG by LCBA‐FACS.

A secondary analysis was performed considering patients fulfilling NMOSD diagnostic criteria based on historical AQP4‐IgG seropositivity who were now negative by both assays (seroreversion) as being “false‐negatives.” Using this method, AQP4‐IgG LCBA‐FACS exhibited 68.6% (95% CI: 59%–77%) sensitivity and 100% (95% CI: 99%–100%) specificity, whereas AQP4‐IgG FCBA‐IF had 50.5% (95% CI: 41%–60%) sensitivity and 100% (95% CI: 99%–100%) specificity (Tables 5 and 6).

Overall, the total number of samples drawn from NMOSD patients within 90 days of an attack was 11 (4 exclusively positive by LCBA‐FACS and 7 positives by both assays). Thus, in the acute setting, AQP4‐IgG LCBA‐FACS had a sensitivity of 100% (95% CI: 72%–100%) (11/11), whereas AQP4‐IgG FCBA‐IF had 63.6% (95% CI: 31%–89%) (7/11) sensitivity.

No differences in clinical characteristics (attack phenotype) or demographic characteristics (age and sex) were observed between patients testing positive by both AQP4‐IgG FCBA‐IF and LCBA‐FACS and patients testing positive by only LCBA‐FACS.

## Discussion

In summary, our study shows that LCBA‐FACS for both MOG‐IgG and AQP4‐IgG had markedly better sensitivity than FCBA‐IF, with similar specificity. Consequently, the use of FCBA‐IF for detection of MOG‐IgG and AQP4‐IgG may result in significant underdiagnosis of both MOGAD and AQP4+ NMOSD. The observed difference was especially stark for MOG‐IgG, with less than half of diagnosed MOGAD cases seropositive by LCBA‐FACS identified by FCBA‐IF.

Notably, while other studies comparing MOG‐IgG FCBA and LCBA have reported somewhat conflicting results, our findings are overall in line with the majority of prior reports. Tea et al reported that only 55% of MOG‐IgG positive sera by LCBA were able to bind to fixed MOG, a finding that is remarkably similar to the results of our study.^12^ Other studies have also reported lower sensitivity of MOG‐IgG FCBA compared to LCBA, although the degrees of discrepancy were more modest compared to our results.^3^, ^6^, ^17^ A recent study from a clinical laboratory reported good agreement between MOG‐IgG FCBA‐IF and LCBA‐FACS (92.9%); however, clinical data were unavailable to determine MOGAD diagnosis (precluding the estimation of diagnostic specificity/sensitivity) and agreement was mainly driven by samples that were negative by both assays (for samples that were positive by at least one assay, agreement was only 70.7% [as calculated by data presented in the manuscript's figures]).^4^

For AQP4‐IgG, a prior comparative multi‐center study assessing multiple assays run at different locations, including by the fixed CBA‐IF kit manufacturer (Euroimmun) found sensitivities for LCBAs ranging from 98.5% to 100%, while for FCBA‐IF performed in‐house by Euroimmun, the range was 92.4%–93.9%, and FCBA‐IF performed at other diagnostic centers had sensitivities between 84.8% and 93.9%.^10^ While fixed CBA‐IF at our center had modestly lower sensitivity, our results are overall in line with this study that found moderately lower sensitivity of AQP4‐IgG FCBA‐IF compared to LCBA. It should be noted, however, that another study found near‐identical results for AQP4‐IgG LCBA versus FCBA‐IF.^9^ In this setting, it is conceivable that differences in laboratory practices and training may account for discrepancies in AQP4‐IgG FCBA‐IF between different studies and centers; however, we emphasize that FCBA‐IF in this study was performed at a large academic referral center laboratory, with extensive expertise and experience in performing immunological assays.^3^, ^7^

The main potential etiology for the worse performance of FCBA compared to LCBA is the effects of fixation with formaldehyde on MOG and AQP4. Formaldehyde fixation alters protein structure and can distort antigenic epitopes, thus disrupting antigen–antibody interactions of MOG‐IgG and AQP4‐IgG.^12^ The fact that the observed discrepancies between FCBA and LCBA are more marked for MOG compared to AQP4 is consistent with this hypothesis, as MOG‐IgG binding is highly sensitive to antigen conformation requiring the use of CBAs with full‐length MOG for detection, in contrast to AQP4‐IgG which can be detected by the use of enzyme‐linked immunosorbent assay (ELISA), albeit with lower specificity/sensitivity compared to CBAs.^8^, ^10^ Additionally, it has been previously reported that MOG‐IgG1 from most MOGAD patients requires bivalent binding.^18^ In this setting, it is conceivable that formaldehyde fixation may also impact interactions of the intracellular part of MOG and affect the distance between MOG monomers, which must be at a suitable distance to support bivalent binding.^18^

Interestingly, there was a small number of MOGAD cases with positive FCBA‐IF and negative LCBA‐FACS. These specimens all had low MOG‐IgG end‐titers by FCBA‐IF (≤1:20). FCBA‐IF employs a screening dilution of 1:10, whereas LCBA‐FACS uses 1:20, which could partially account for these observed differences.^13^ Additionally, the LCBA‐FACS assay used in this study specifically tests for MOG‐IgG1, while FCBA‐IF assesses all IgG subclasses, suggesting that, in low‐titer seropositive cases, the possible limited presence of MOG‐IgG1 (and presence of other IgG subclasses) may have contributed to false‐negative outcomes by LCBA.^3^, ^7^, ^19^, ^20^

Furthermore, it is important to highlight that among those who were MOG‐IgG seropositive by both assays, all of whom met diagnostic criteria for MOGAD, only 9% were clear‐positive by FCBA‐IF, compared to 73% for LCBA‐FACS. Furthermore, FCBA‐IF exhibited excellent specificity in this study. Notably, the 1:100 cut‐off for FCBA‐IF proposed in the 2023 MOGAD diagnostic criteria has not been formally validated and has been based on the manufacturer's instructions to test at a screening dilution of 1:10 and then perform serial 10‐fold dilutions for titration.^1^, ^13^ However, we and others employ a serial twofold dilution approach for titration of MOG‐IgG CBA‐IF, which allows for the determination of intermediate titers between 1:10 and 1:100. Our findings support that the 1:100 cut‐off for clear‐positive by FCBA‐IF could likely be lower, and additional studies validating specific cut‐offs are needed.

Failure to make an accurate diagnosis of MOGAD or AQP4+ NMOSD has important implications, as this adversely impacts a clinician's ability to prognosticate, appropriately counsel patients, implement effective therapy that is specific for the underlying disease, or enroll in clinical trials. While the discrepancy in sensitivity between FCBA‐IF and LCBA‐FACS was less marked for AQP4‐IgG, a missed diagnosis of AQP4+ NMOSD is especially consequential, given the availability of highly effective targeted therapies for AQP4+ NMOSD and the high risk of permanent neurological disability, even with a single attack.^21^ These discrepancies in CBA sensitivity also need to be carefully considered in research on “double‐seronegative” NMOSD (i.e., negative for both AQP4‐IgG and MOG‐IgG), since it is expected that studies predominantly using FCBAs may include a significant number of patients with false‐negative auto‐antibody testing. Notably, even with the use of LCBA, false‐negative results can still occur due to issues related to the timing of collection (especially in MOGAD where spontaneous seroreversion is common), specimen type (serum versus CSF) and effects of treatment.^15^, ^17^, ^22^, ^23^, ^24^

In this study, specificity was excellent for AQP4‐IgG with both assays, with no false‐positive results identified. For MOG‐IgG, specificity was slightly lower with LCBA‐FACS, mainly reflecting false‐positives at low titer and highlighting the need for cautious interpretation of low MOG‐IgG titers in patients with atypical clinical phenotypes.^25^, ^26^ The high specificity of FCBA‐IF found in our study makes it suitable for initial screening in resource‐limited settings. However, when clinical suspicion is high, caution is required due to the increased risk of false‐negatives. In such cases, LCBA should be considered to ensure diagnostic accuracy.

Strengths of this study include the large sample size, availability of detailed clinical information of participants, and testing of specimens in a real‐world setting consistent with routine clinical practice. Limitations include the retrospective nature of the study, leading to the following: (1) sampling performed at variable timepoints in the clinical course (e.g., disease onset, relapse or remission; with or without use of immunosuppressive therapy); (2) testing by both FCBA and LCBA not performed for all patients with suspected demyelinating attacks (although this would not be expected to significantly impact estimates of specificity/sensitivity, in contrast to positive/negative predictive value which are dependent on the disease prevalence in the studied population); (3) use of archived frozen serum specimens in a subset of cases. Another limitation of our study is that the analysis was performed in a single center. Variability in FCBA sensitivity across sites may reflect differences in laboratory techniques and practices, which highlights the need for training and standardization of these assays across centers. Finally, our study was limited to evaluation of serum. While CSF AQP4‐IgG testing has limited utility, CSF MOG‐IgG testing may identify patients with a MOGAD phenotype who are MOG‐IgG seronegative, and studies comparing the performance of CSF MOG‐IgG LCBA and FCBA are needed.^22^, ^23^, ^24^, ^27^

In conclusion, our study supports that the use of FCBAs may result in underdiagnosis of both MOGAD and AQP4 + NMOSD. Future efforts should focus on consensus guidelines for MOG‐IgG and AQP4‐IgG testing and the development of standards for comparisons of assay titers and performance.

## Author Contributions

Conception and design of the study: PC, SJP, EPF, and ESS. Acquisition, analysis, and interpretation of data: YS, AF, CT, LR, KG, MDS, YR, JJC, PAC, PC, SJP, EPF, and ESS. Drafting and revision of the manuscript for intellectual content: YS, AF, CT, LR, KG, MDS, YR, JJC, PAC, PC, SJP, EPF, and ESS.

## Funding Information

This study was funded by the Caring Friends for NMO Research Fund, the Mayo Clinic Center for MS and Autoimmune Neurology, and the National Institutes of Health (R01NS113828 to EPF and K23NS117883 to ESS).

## Conflicts of Interest

Y. Said, A. Filippatou, C. Tran, L. Rezavi, M. Caturegli, Y. Resto, and M. Smith report no disclosures. K. Guo works in the Mayo Clinic Neuroimmunology Laboratory. J. Chen has received personal compensation for serving as a consultant for UCB and Horizon. P. Calabresi reports financial support provided by the National Institute of Neurological Disorders and Stroke. Pr. Calabresi has received grants from the Myelin Repair Foundation, the US Department of Defense, the National Multiple Sclerosis Society, and Genentech. Pr. Calabresi has been consulted for Idorsia Pharmaceuticals Ltd and Eli Lilly and Company. Pr. Calabresi is also a scientific advisor to Spolia Therapeutics. S. J. Pittock has received personal compensation for serving as a consultant for Roche/Genentech, Sage Therapeutics, Astellas, and Arialis. He has received personal compensation for serving on scientific advisory boards or data safety monitoring boards for F. Hoffman‐La Roche AG, Genentech, and UCB. His institution has received compensation for serving as a consultant for Astellas, Alexion/AstraZeneca Rare Diseases, and Horizon/Amgen. All compensation is paid to Mayo Clinic. He has received research support from Alexion/AstraZeneca Rare Diseases, Horizon/Amgen, and F. Hoffman‐La Roche AG, Genentech. He has a patent, Patent# 8,889,102 (Application#12‐678350, Neuromyelitis Optica Autoantibodies as a Marker for Neoplasia) – issued; a patent, Patent# 9,891,219B2 (Application#12‐573942, Methods for Treating Neuromyelitis Optica (NMO) by administration of eculizumab to an individual that is aquaporin‐4 (AQP4)‐IgG autoantibody positive) – issued and from which he has received royalties. He is working as a consultant in the Mayo Clinic Neuroimmunology laboratory clinical service. E. Flanagan has served on advisory boards for Alexion, Genentech, Horizon Therapeutics, and UCB. He has received research support from UCB. He received royalties from UpToDate. Dr. Flanagan is a site principal investigator in a randomized clinical trial of rozanolixizumab for relapsing myelin oligodendrocyte glycoprotein antibody‐associated disease run by UCB. Dr. Flanagan is a site principal investigator and a member of the steering committee for a clinical trial of satralizumab for relapsing myelin oligodendrocyte glycoprotein antibody‐associated disease run by Roche/Genentech. Dr. Flanagan has received funding from the NIH (R01NS113828). Dr. Flanagan is a member of the medical advisory board of the MOG project. Dr. Flanagan is an editorial board member of Neurology, Neuroimmunology and Neuroinflammation, the Journal of the Neurological Sciences, and Neuroimmunology Reports. A patent has been submitted on DACH1‐IgG as a biomarker of paraneoplastic autoimmunity. E. Sotirchos has consulted for Alexion, Horizon Therapeutics, Amgen, TG Therapeutics, and Roche/Genentech, and is a site principal investigator for clinical trials funded by Roche/Genentech and UCB.

## Supporting information


**Figure S1.** Comparison of MOG‐IgG titers: Fixed CBA versus live CBA for MOG‐IgG seropositive patients fulfilling 2023 MOGAD diagnostic criteria. *FCBA‐IF, fixed cell‐based assay using immunofluorescence; IgG, immunoglobulin; LCBA‐FACS, live cell‐based assay using flow cytometry. The red dashed line in the MOG‐IgG titer Figure* 2A *represents the cut‐offs for clear‐positive versus low‐positive titers (1:100 dilution for both assays).*

**Table S1.** Comparison of MOG‐IgG results between FCBA‐IF and LCBA‐FACS using clinical and frozen samples.

**Table S2.** Comparison of AQP4‐IgG results between FCBA‐IF and LCBA‐FACS using clinical and frozen samples.

**Table S3.** Case summaries and diagnoses for patients with discrepant MOG‐IgG FCBA‐IF and LCBA‐FACS not fulfilling 2023 MOGAD Diagnostic Criteria. *CNS, central nervous system; CSF, cerebrospinal fluid; IgG, immunoglobulin G; IgM, immunoglobulin M; MS, multiple sclerosis; OCB, oligoclonal bands; ON, optic neuritis; RRMS, relapsing‐remitting multiple sclerosis; TM, transverse myelitis.*

## Data Availability

Anonymized data used for this study are available from the corresponding author on reasonable request, with the proper data‐sharing agreements in place.
