# Supplementary figures and images for: Real‐world clinical experience with serum MOG and AQP4 antibody testing by live versus fixed cell‐based assay
Source: Ann Clin Transl Neurol. 2025 Feb 3;12(3):556–64. doi: 10.1002/acn3.52310 (PMC11920744; doi:10.1002/acn3.52310)

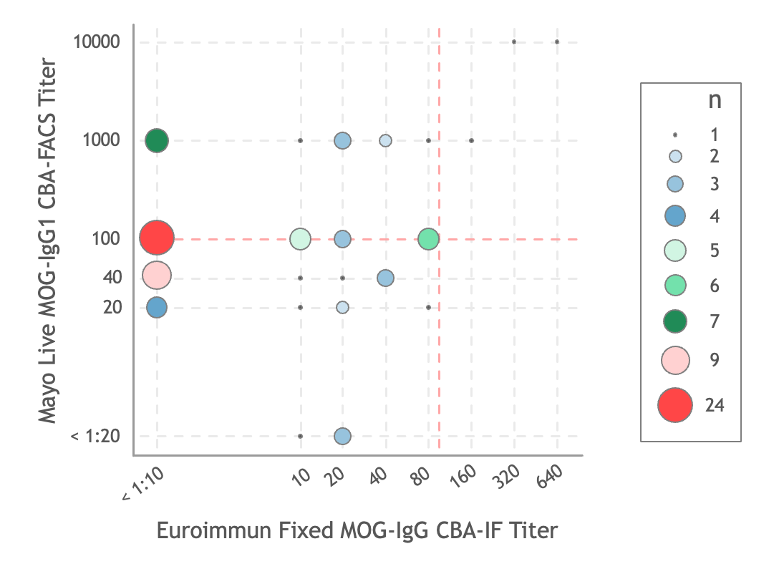

Supplement: Supplementary file 1 — Figure S1. Comparison of MOG‐IgG titers: Fixed CBA versus live CBA for MOG‐IgG seropositive patients fulfilling 2023 MOGAD diagnostic criteria. FCBA‐IF, fixed cell‐based assay using immunofluorescence; IgG, immunoglobulin; LCBA‐FACS, live cell‐based assay using flow cytometry. The red dashed line in the MOG‐IgG titer Figure 2A represents the cut‐offs for clear‐positive versus low‐positive titers (1:100 dilution for both assays). [file ACN3-12-556-s004.png]
