# Supplementary material for: Real‐world clinical experience with serum MOG and AQP4 antibody testing by live versus fixed cell‐based assay
Source: Ann Clin Transl Neurol. 2025 Feb 3;12(3):556–64. doi: 10.1002/acn3.52310 (PMC11920744; doi:10.1002/acn3.52310)
Supplement: Supplementary file 2 — Table S1. Comparison of MOG‐IgG results between FCBA‐IF and LCBA‐FACS using clinical and frozen samples. [file ACN3-12-556-s001.docx]

**Supplementary Table 1:** Comparison of MOG-IgG results between FCBA-IF and LCBA-FACS using clinical and frozen samples

|  |  |  |  |  |  |
| --- | --- | --- | --- | --- | --- |
|  |  |  | **MOG-IgG LCBA FACS** | |  |
|  |  |  | **Positive** | **Negative** | **Total** |
| **MOG-IgG FCBA IF** | **Positive** | Clinical samples | 17 | 1 | 18 |
|  |  | Frozen samples | 16 | 4 | 20 |
|  |  | Total | 33 | 5 | 38 |
|  | **Negative** | Clinical samples | 45 | 489 | 534 |
|  |  | Frozen samples | 11 | 11 | 22 |
|  |  | Total | 56 | 500 | 556 |
|  |  | **Total** | 89 | 505 | 594 |
