# Supplementary material for: Real‐world clinical experience with serum MOG and AQP4 antibody testing by live versus fixed cell‐based assay
Source: Ann Clin Transl Neurol. 2025 Feb 3;12(3):556–64. doi: 10.1002/acn3.52310 (PMC11920744; doi:10.1002/acn3.52310)
Supplement: Supplementary file 3 — Table S2. Comparison of AQP4‐IgG results between FCBA‐IF and LCBA‐FACS using clinical and frozen samples. [file ACN3-12-556-s002.docx]

**Supplementary Table 2:** Comparison of AQP4-IgG results between FCBA-IF and LCBA-FACS using clinical and frozen samples

|  |  |  | **AQP4-IgG LCBA FACS** | |  |
| --- | --- | --- | --- | --- | --- |
|  |  |  | **Positive** | **Negative** | **Total** |
| **AQP4-IgG FCBA IF** | **Positive** | Clinical samples | 9 | 1 | 10 |
|  |  | Frozen samples | 42 | 1 | 43 |
|  |  | Total | 51 | 2 | 53 |
|  | **Negative** | Clinical samples | 12 | 487 | 499 |
|  |  | Frozen samples | 9 | 16 | 25 |
|  |  | Total | 21 | 503 | 524 |
|  |  | **Total** | 72 | 505 | 577 |
