# Supplementary material for: Real‐world clinical experience with serum MOG and AQP4 antibody testing by live versus fixed cell‐based assay
Source: Ann Clin Transl Neurol. 2025 Feb 3;12(3):556–64. doi: 10.1002/acn3.52310 (PMC11920744; doi:10.1002/acn3.52310)
Supplement: Supplementary file 4 — Table S3. Case summaries and diagnoses for patients with discrepant MOG‐IgG FCBA‐IF and LCBA‐FACS not fulfilling 2023 MOGAD Diagnostic Criteria. CNS, central nervous system; CSF, cerebrospinal fluid; IgG, immunoglobulin G; IgM, immunoglobulin M; MS, multiple sclerosis; OCB, oligoclonal bands; ON, optic neuritis; RRMS, relapsing‐remitting multiple sclerosis; TM, transverse myelitis. [file ACN3-12-556-s003.docx]

**Supplementary table 3**: Case summaries and diagnoses for patients with discrepant MOG-IgG FCBA-IF and LCBA-FACS not fulfilling 2023 MOGAD Diagnostic Criteria

| **Case Summary** | **MOG-IgG FCBA-IF Result** | **MOG-IgG LCBA-FACS Result** |
| --- | --- | --- |
| Clinically isolated syndrome (short-segment TM with eccentrically located, small focal posterolateral thoracic lesion; no CSF-specific OCB; no additional CNS lesions) | Negative | Positive  1:20 |
| RRMS (short-segment ON without peri-neuritis or optic disc swelling, accrued additional asymptomatic CNS lesions over time with appearance typical for MS, CSF-specific OCB) | Negative | Positive  1:40 |
| RRMS (short-segment ON without peri-neuritis or optic disc swelling, CNS lesions with appearance typical for MS, CSF-specific OCB) | Negative | Positive  1:40 |
| RRMS (brainstem attack with bilateral internuclear ophthalmoplegia and post-gadolinium enhancing pontine lesion; additional CNS lesions with appearance typical for MS, CSF-specific OCB) | Negative | Positive  1:100 |
| SPMS (progressive lower extremity weakness, remote attacks including ON, CNS lesions with appearance typical for MS) | Negative | Positive  1:40 |
| Radiologically isolated syndrome (non-specific neurological symptoms, no clear history of a clinical demyelinating event; CNS lesions with appearance typical for MS; CSF-specific OCB) | Negative | Positive  1:100 |
| Radiologically isolated syndrome (non-specific neurological symptoms, no clear history of a clinical demyelinating event; CNS lesions with appearance typical for MS; CSF analysis not performed) | Negative | Positive  1:100 |
| Migraine (non-specific neurological symptoms, no clear history of a clinical demyelinating event, non-specific neurologic symptoms, history of migraines; MRI brain with non-specific white matter changes) | Negative | Positive  1:40 |
| Viral meningitis (headaches, gastrointestinal symptoms, fever and altered mental status, known rodent exposure; MRI brain with diffuse leptomeningeal enhancement without parenchymal abnormality, positive lymphocytic choriomeningitis virus [LCMV] by metagenomic next-generation sequencing in CSF and positive CSF LCMV IgG/IgM) | Negative | Positive  1:100 |
| Metabolic optic neuropathy (bilateral slowly progressive vision changes; MRI orbits without focal optic nerve T2 lesions or post-gadolinium enhancement; history of heavy alcohol use with multiple nutritional deficiencies including vitamin B12, B1, folate, and zinc) | Negative | Positive  1:20 |
| Giant cell arteritis (temporal artery biopsy-proven giant cell arteritis with presentation consistent with unilateral arteritic anterior ischemic optic neuropathy) | Negative | Positive  1:20 |
| Epidural lipomatosis (chronic progressive myelopathy, non-enhancing thoracic longitudinally extensive spinal cord lesion associated with marked epidural lipomatosis) | Negative | Positive  1:20 |
| Functional vision loss (headache and sequential vision loss in setting of morbid obesity; no optic disc swelling, normal CSF opening pressure; MRI brain and orbits, MR venography, visual evoked potentials, full-field electroretinography, optical coherence tomography, fluorescein angiography, and fundus autofluorescence were all unremarkable; | Positive  1:20 | Negative |

*TM: Transverse myelitis; CSF: Cerebrospinal fluid; OCB: Oligoclonal bands; CNS: Central nervous system; RRMS: Relapsing-Remitting Multiple Sclerosis; ON: Optic neuritis; MS: Multiple Sclerosis; IgG: Immunoglobulin G; IgM: Immunoglobulin M.*
